# Learning curve of robotic assisted microsurgery in surgeons with different skill levels: a prospective preclinical study

**DOI:** 10.1007/s11701-024-02114-2

**Published:** 2024-09-28

**Authors:** Donata von Reibnitz, Andrea Weinzierl, Lisanne Grünherz, Pietro Giovanoli, Nicole Lindenblatt

**Affiliations:** 1 Department of Plastic and Hand Surgery, University Hospital Zurich (USZ), Zurich, Switzerland; 2 University of Zurich (UZH), Zurich, Switzerland

**Keywords:** Microsurgery, Robotic assisted microsurgery, Surgical robotic system, Microsurgery training, Microsurgery learning curve

## Abstract

Achieving precision in microsurgery requires skill, adequate instruments and magnification, as well as extensive training. Dedicated surgical robotic systems have enhanced and expanded the application of (super-)microsurgical techniques by introducing motion scaling and providing improved surgeon ergonomics. In this prospective preclinical trial, we analyzed the learning curve in robotic assisted microsurgery in 13 participants including medical students, residents, and attending physicians. Data on demographics as well as prior experience in surgery, microsurgery, and gaming were collected. In three study sessions, the participants performed nine microsurgical anastomoses each on 2 mm vessel models using the Symani^®^ Surgical System in combination the VITOM 3D exoscope. A senior expert microsurgeon reviewed the de-identified and blinded videos and scored all anastomoses using a modified “Structured Assessment of Microsurgical Skills” (SARMS) score. All participants significantly reduced their time needed per anastomosis and their overall SARMS score, as well as individual scores for motion and speed throughout the trial. We saw a significant correlation of prior years of practice in surgery with the overall mean time and mean SARMS score. In a separate analysis of the three sessions, this influence could no longer be seen in the last session. Furthermore, we found no significant effect of gender, age, hand dominance, or gaming experience on speed and quality of the anastomoses. In this study of 117 robotic assisted anastomoses, a rapid improvement of performance of all participants with different surgical skills levels could be shown, serving as encouraging evidence for further research in the implementation of microsurgical robotic systems.

**Supplementary Information:**

The online version contains supplementary material available at 10.1007/s11701-024-02114-2.

## Introduction

Microsurgery is a complex skill requiring a high level of precision and enhanced visualization. Aided by the development of improved intraoperative magnification with digital microscope systems, the field has since evolved to encompass supermicrosurgery, which is defined as the manipulation on structures measuring less than 1 mm in diameter, commonly 0.3–0.8 mm [1]. In addition to surgical microscopes or loupes, special microsurgical instruments are needed for handling the delicate anatomical structures. Challenges in microsurgery include the demand in steadiness, exceptional hand-eye coordination and persistent concentration, resulting in a high physical and mental strain on the surgeon, as well as the necessary extensive training to reach sufficient levels of proficiency in this skill.

Robotic assistance in surgery, though first introduced in the field of endoscopic surgery with the Da Vinci Surgical System (Intuitive Surgical Inc., Sunnyvale, USA), has gained traction in microsurgery in recent years, addressing the issues of surgeon ergonomics and tremor reduction through motion scaling. Significant advances have been made possible by the introduction of two designated microsurgical robotic systems, namely MUSA MicroSure (MicroSure, Eindhoven, The Netherlands) and the Symani Surgical System (Medical Microinstruments Inc., Wilmington, Del., USA). Since their establishment in 2020 and 2021, respectively, indications for robotic assisted microsurgery have considerably increased and data are now available on the use of robotic assistance in a plethora of (super-)microsurgical settings including lymphatic reconstruction [2–7], head and neck, extremity, and breast reconstruction [8–17], central and peripheral nerve surgery [14, 18, 19], and ophthalmology [20].

Routine implementation of robotic assisted microsurgery has so far been restrained by high material costs, lack of cost-effectiveness data, complex instruction needed for surgical team and assumed shallow and prolonged learning curve for unexperienced surgeons. This study aims to understand the learning curve involved in acquiring the skill set needed to perform robot-assisted microsurgical anastomoses with the Symani Surgical System across a heterogeneous study cohort with different pre-study surgical skill levels.

## Materials and methods

The study was a single-center prospective preclinical trial. The study took place at the University Hospital of Zurich in Zurich, Switzerland. A clarification of responsibility of the Zurich Cantonal Ethics Committee was requested (BASEC Nr. Req. 2022–01239) and an authorization waiver was granted; no separate approval was thus required.

### Participants

The participants were recruited from our department of plastic and hand surgery and included attending physicians, residents and medical students. The participants had no prior experience using the surgical robotic system. All participants filled out a pre-study questionnaire that included questions regarding demographics, prior (micro-) surgical and surgical experience and self-rated experience level in microsurgery (loupes/microscope), robotic surgery, laparoscopic surgery and gaming (PC/console). Experience levels were graded 1 (novice/no experience), 2 (beginner), 3 (advanced), and 4 (expert).

### Experimental setup

**Fig. 1:**
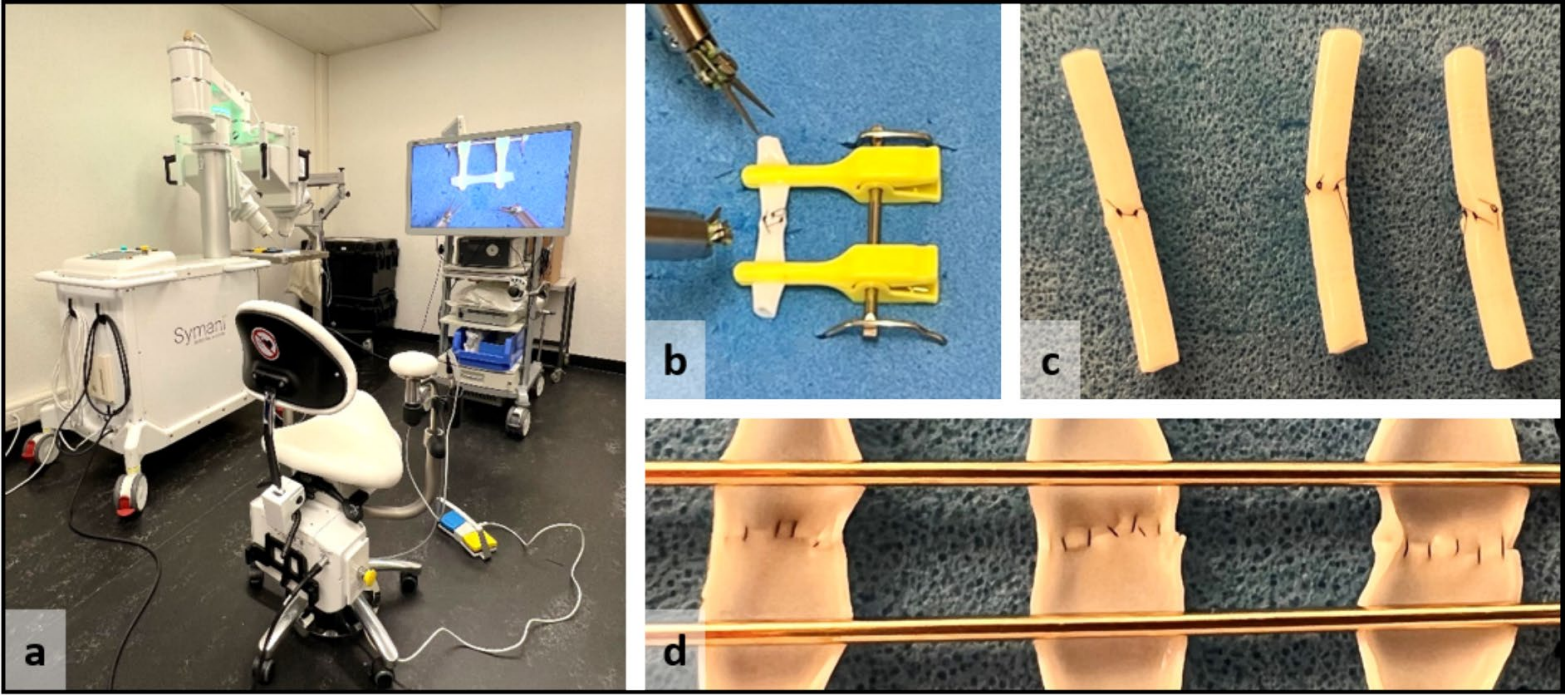
**a** Experimental setup consisting of Symani Surgical System and VITOM 3D exoscope system. The surgeon console consisting of an ergonomically designed chair, in which the participant sits. Attached to the chair are the manipulators and the footswitch, with which the participant is able to control the instrumentation. **b** Setup of 2 mm PCA vessel fixed with double microvascular clamp. **c** Completed set of three anastomosis before and d after dissection for inspection

The robotic system used for this study was the Symani Surgical System (Medical Microinstruments, Inc., Wilmington, USA). The setup of this system includes the portable cart, which includes the macropositioner and the micromanipulators (Fig. 1a). The surgeon console consists of an ergonomically designed chair, in which the participant sits and holds the manipulators. The participant is able to further control the instrumentation via footswitch [21]. For this study, we used the micro-dilator and micro-needle holder suture cut NanoWrist^®^ Instruments. Motion scaling was set to x10.

Visualization was performed using the VITOM 3D exoscope system (Karl Storz SE & Co. KG, Tuttlingen, Germany). The VITOM 3D camera was positioned at a fixed interval above the experimental setup. The participant wore 3D polarized glasses and was seated in front of the 32” 3D/4K display in the Symani surgeon console. Chair position and distance to the monitor was adjusted to the participants’ sight and preferences.

The anastomoses were performed on artificial vessels made from PVA (polyvinyl alcohol) with 2 mm diameter (WetLab Corporation, Japan) using 8–0 nylon microsurgical sutures with a 3/8 circle needle (BEAR Medic Corporation, Japan). The vessels were fixed on a foam rubber surface using a double microvascular clamp (TKM-2, Biover, Hergiswil, Switzerland) (Fig. 1b).

### Study design

All participants received a short introduction to the system and performed three training exercises before starting the study sessions. The study itself consisted of three separate sessions, a minimum of 4 and a maximum of 10 days apart. At the beginning of the first session, participants received a brief instruction regarding the study setup and started with a training anastomosis, for which they performed three sutures on the test vessel. During each session, participants then performed three anastomoses each, totaling nine anastomoses with six sutures each. The vessels were prepared, cut and fixed according to protocol by the study assistant. Per anastomosis, the participant received one 8-0 suture of 12.5 cm length. The participant performed three sutures each on the front and the backside of the vessel. The study assistant turned the vessel 180° manually after the first three sutures and kept the vessels moist during this time. Sutures were tied with one double throw and one single throw. Sutures were cut by the participants themselves using the needle holder suture cut instrument. Time per anastomosis was recorded starting as soon as the participant picked up the suture with an instrument and finishing as soon as the last suture was cut, pausing in between for the flipping of the vessel. The study assistant recorded all problems during the anastomosis (i.e., accidental cutting/tearing of the vessel or the suture material, tearing of the anastomosis, loss of suture material, need for additional suture material, and damage of instruments). The procedure was repeated for each session except for the training anastomosis, which was only performed at the beginning of the first session.

The vessels were dissected after completion of the anastomosis and the sutures checked for back wall stiches. All anastomoses were video-recorded. The videos were then de-identified and blinded regarding participant and session/anastomosis and were reviewed by a single experienced microsurgeon at our institution. Each anastomosis was graded according to the Structured Assessment of Microsurgical Skills (SARMS) [22, 23] scoring system. We slightly modified the original score, proposed by Liverneaux et al. to better fit our study design. The performance level in ten skills across the categories dexterity, visuo-spatial ability, operative flow, and robotic skill was rated on a scale from one to five, with five being the highest score. The details of the score system can be found in Additional Material 1.

### Statistical analysis

The statistical analysis was performed using GraphPad Prism, version 10.2.0 (GraphPad, Massachusetts, USA). Descriptive statistics were calculated for all demographic and training level/experience data. Time, SARMS score (individual and combined) and problems per anastomosis were evaluated and means calculated. A comparison of these parameters per session was performed using repeated-measures one-way ANOVA and paired t-test. The influence of demographics as well as training level/experience on mean time, score and problems as well as their difference between first and third sessions was analyzed using one-way ANOVA and unpaired t-test for categorical values and correlation analysis for continuous variables. A *p* value of 0.05 or lower was considered statistically significant.

## Results

In total, 13 participants took part in this study, performing nine anastomoses each, thus leading to a total of 117 robotic assisted microsurgical anastomoses for analysis. Participants included three medical students and junior residents with 2 or less years of experience, seven residents, two junior attendings with 3 or less years of experience and one senior attending (more than 3 years of experience). The participants had between 0 and 11 years of practice in surgery (mean 3.7 years) and 0–10 years of practice in microsurgery (mean 1.6 years). Further demographic data and level of experience in different categories can be found in Table 1.

**Table 1:** Participant demographic data and level of experience in different skills (*n* = 13)

Years of practice in surgery	Mean 3.7 years, range 0–11 years			
Years of practice in microsurgery	Mean 1.6 years, range 0–10 years			
Level of training	*Medical student/junior resident*	*Senior resident*	*Junior attending*	*Senior attending*
	*n* = 3	*n* = 7	*n* = 2	*n* = 1
Level of expertise in microsurgery with loupe	*Level 1*	*Level 2*	*Level 3*	*Level 4*
	*n* = 4	*n* = 6	*n* = 2	*n* = 1
Level of expertise in microsurgery with microscope	*Level 1*	*Level 2*	*Level 3*	*Level 4*
	*n* = 5	*n* = 5	*n* = 2	*n* = 1
Level of expertise in laparoscopy	*Level 1*	*Level 2*	*Level 3*	*Level 4*
	*n* = 5	*n* = 8		
Level of expertise in robotic surgery	*Level 1*	*Level 2*	*Level 3*	*Level 4*
	*n* = 10	*n* = 3		
Number of microsurgical sutures performed with a microscope in the past 12 months	*0 to 5*	*6 to 20*	*more than 20*	
	*n* = 8	*n* = 4	*n* = 1	
Number of microsurgical sutures performed with a loupe in the past 12 months	*0 to 5*	*6 to 20*	*more than 20*	
	*n* = 11	*n* = 1	*n* = 1	
Gaming—level of expertise in PC gaming	*Level 1*	*Level 2*	*Level 3*	*Level 4*
	*n* = 5	*n* = 6	*n* = 1	*n* = 1
Gaming—level of expertise in console gaming (joystick-based)	*Level 1*	*Level 2*	*Level 3*	*Level 4*
	*n* = 3	*n* = 6	*n* = 3	*n* = 1
Age (in years)	*18–25*	*26–30*	*31–35*	*36–40*
	*n* = 1	*n* = 5	*n* = 4	*n* = 3
Dominant hand	*Right*	*Left*		
	*n* = 12	*n* = 1		
Gender	*Female*	*Male*		
	*n* = 6	*n* = 7		

Medical student/junior resident: ≤ 2-year experience, senior resident: > 2-year experience, junior attending: ≤ 3-year experience, senior attending: > 3-year experience. Level 1: novice/no experience, Level 2: beginner, Level 3: advanced, Level 4: expert

**Fig. 2:**
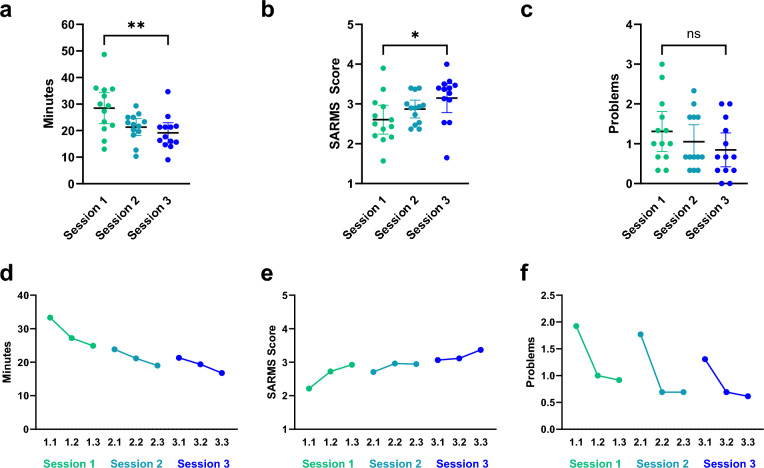
**a** Mean time, **b** mean SARMS score, and **c** mean number of problems per anastomosis in session 1–3 (*n* = 13, three anastomoses per session). Each dot represents the mean of one participant per session. Paired t-test for comparison of means between session 1 and 3. **d** Mean time, **e** mean SARMS score, and **f** mean number of problems of each anastomosis, grouped by session. Error bar: mean with 95% confidence interval (CI). **p value ≤ 0.01, *p value ≤ 0.05, ns *p* value > 0.05

The means of time per anastomosis, SARMS score (mean of all ten score categories) and problems encountered were calculated for session one, two, and three, respectively. The mean time per anastomosis across all participants was 28.5, 21.3, and 19.3 minutes; the mean SARMS score per anastomosis 2.6, 2.9, and 3.1; and the mean number of problems encountered 1.3, 1.1 and 0.8, respectively. In total, 123 problems were recorded. The most common problem was accidental cutting or tearing of the suture (*n* = 59) requiring more than one suture material per anastomosis in 14 cases, followed by loss of suture (*n* = 28), damage of the vessel (*n* = 18), and damage of material (*n* = 3). One case of back wall stitching was noted. Repeated measures ANOVA showed a significant difference between the mean time per anastomosis and the mean SARMS score across the three sessions (*p* = 0.001, *p* = 0.032, respectively), but no significant difference of the mean number of problems per anastomosis (*p* = 0.179). Figure 2a–c shows the means per session for all categories with paired t-test for comparison between session 1 and 3. In Fig. 2d–f, the improvement of time, score and reduction of problems during each session can be seen.

**Fig. 3:**
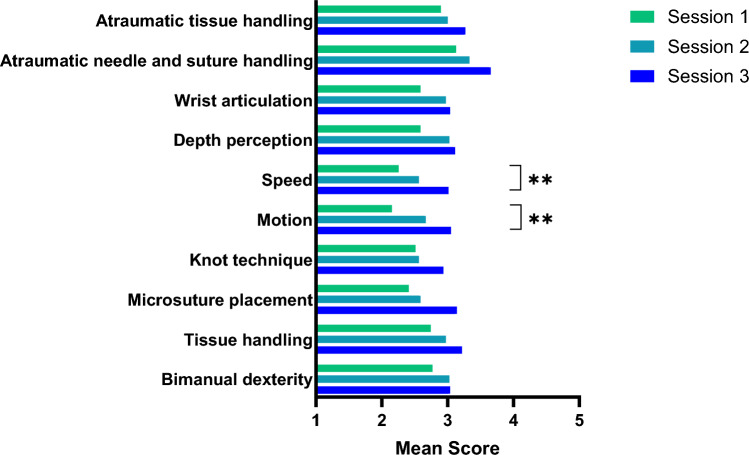
Mean SARMS category scores in session 1 to 3 (*n* = 13, three anastomoses per session). Paired t-test for comparison of means between sessions 1 and 3. **p value ≤ 0.01, *p value ≤ 0.05, ns *p* value > 0.05

All SARMS score categories were analyzed separately and paired t-test was performed to compare means of the first and third sessions. Mean scores in all categories improved with every session across the whole group of participants (Fig. 3). The improvement in the categories motion and speed was statistically significant with *p* = 0.003 and *p* = 0.007, respectively.

**Fig. 4:**
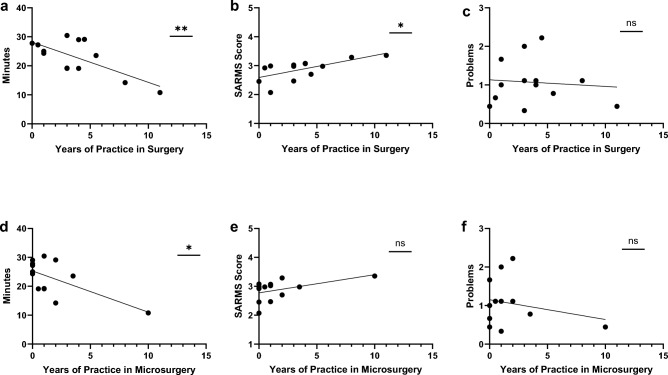
Correlation analysis of years of experience in surgery and microsurgery with mean time (**a**, **d**), mean SARMS score (**b**, **e**), and mean problems (**c**, **f**) per anastomosis. **p value ≤ 0.01, *p value ≤ 0.05, ns *p* value > 0.05

We performed a correlation analysis to examine the influence of experience in surgery and microsurgery on time, score and problems (Fig. 4). The number of years of practice in surgery was significantly correlated with the mean time (Pearson *r* = −0.706, *p* = 0.007) as well as the mean SARMS score (*r* = 0.657, *p* = 0.015) across all the sessions. The number of years of practice in microsurgery were only correlated significantly with the mean time (*r* = −0.638, *p* = 0.019), not with the mean SARMS score. When looking at the individual session, we saw a significant correlation of experience in surgery with the mean time in session 1 and 2 (*r* = −0.595, *p* = 0.032; *r* = −0.713, *p* = 0.006) as well as the mean score of session 1 (*r* = 0.559, *p* = 0.047). Experience in microsurgery showed a correlation with mean time of session 2 (*r* = −0.684, *p* = 0.01) and mean score of session 1 (*r* = 0.587, *p* = 0.035). A correlation was neither seen between years of practice in surgery or microsurgery for the difference in time or score between the first and third sessions (Δ time, Δ score), nor for the mean number of problems or Δ problems between the first and the third sessions.

Using ANOVA, we analyzed the relationship between the level of trainings or experience and age on mean time, score and problems as well as Δ time, Δ score and Δ problems. An unpaired t-test was used for those variables where the distribution was only in two categories (level of experience in laparoscopy and robotic surgery, hand dominance, and gender). A higher number of microsurgical sutures performed under a microscope in the past 12 months was significantly associated with a faster mean time (*p* = 0.003). A larger Δ time, meaning a larger improvement along the sessions, was seen in participants that had performed more microsurgical sutures with a loupe (*p* = 0.004). For those who had prior experience in robotic surgery, Δ time was lower than in the group of participants with no prior experience in this field (*p* = 0.036). No significant associations were found for mean score or Δ score. Participants who had performed more microsurgical sutures with a loupe in the past 12 months showed a larger reduction of problems (Δ problems) from session 1 to 3 (*p* = 0.041). The level of training itself was not significantly associated with a difference in mean time, score or problems. Similarly, no significant associations were seen for age, gender, hand dominance or experience in gaming or laparoscopic surgery.

## Discussion

In this study, we analyzed the learning curve of 13 participants in robotic assisted microsurgery in a training environment. We were able to demonstrate that participants of all levels, even with little to no experience in microsurgery, could rapidly improve their speed and skill at robotic assisted microsurgical anastomoses. We saw a significant decrease in time needed per anastomosis across the whole participant cohort after three sessions of three anastomoses each. This is in line with previous studies of robotic assisted microsurgery in preclinical [24–28] and clinical [2, 4, 29] settings. Using the SARMS score, we were able to show that participants could improve on all aspects of evaluation of an anastomosis but especially so in motion and speed. These findings support the hypothesis that training with the robotic surgical system quickly enhances technical abilities in handling the robot while microsurgical skills by themselves require a more intensive training for an equivalent improvement.

The analysis of possible factors influencing the skill and speed of improvement at robotic microsurgical anastomoses revealed a significant association of the years of practice in surgery with the overall mean time and mean SARMS score per anastomosis. This is understandable as the skill of performing a suture is fundamental in all surgical fields. Similarly, participants who had performed more microsurgical sutures with the microscope in the past 12 months and those who had more years of microsurgical experience required less overall time for their anastomoses. In a similar setup, Wessel et al. reported the same effect on time per anastomosis [26].

Interestingly, when looking at the three study sessions separately, a correlation between experience in surgery or microsurgery was only seen in the first and/or second session for time and SARMS score. A possible interpretation of these results is that by the third session, the influence of previous surgical experience on the skill set has declined, thus leveling out the performance of participants across all prior skill levels. Similar results have been reported in robotic surgery simulation, showing that objectively assessed robotic assisted surgery skills are not necessarily dependent on prior experience in clinical applications [30]. A recent meta-analysis of robot-assisted training studies showed that while laparoscopic surgery experience had a positive influence on robotic surgery performance and vice versa, no effect was seen for prior open surgery experience [31]. In our cohort, we saw that while prior robotic experience had no effect on overall time or score, those with some previous contact in robotic surgery had a less steep learning curve (lower Δ time) compared to “robotic-naive” participants.

The number of problems encountered, especially accidental cutting or tearing of the suture, was not correlated with prior surgical or microsurgical experience. This may be explained by the nature of these issues, which were commonly observed to occur due to strangling of the suture around the instruments or the application of undue force, both an aspect of technical handling and controlling of the robotic instruments. Suture tearing, along with vessel damage and damage of the instrument, is likely a result of the lack of haptic feedback, a significant disadvantage in robotic assisted microsurgery. In contrast, motion-scaling aids in improving precision of movements while suturing, especially for novice users, compared to manual anastomoses [26].

Experience in video gaming has been reported to improve performance in robotic assisted surgery, especially in a training environment [32, 33]. Our data did not show any association of self-reported PC and console gaming skill level on anastomosis performance with robotic assistance. Furthermore, no correlation with time, score or number of problems encountered could be seen for age, gender or hand dominance of the participants. Unlike laparoscopy, where instrument grip size and ergonomics pose a potential disadvantage to surgeons of smaller height or hand size [34], robotic setups seem to improve surgeon ergonomics for all users both in surgical [35] as well as microsurgical settings [27, 29].

The statistical analysis of the study data may be limited by the small number of participants per surgical experience level (medical students, junior/senior residents/attendings) but the total number of 117 anastomoses provides a solid foundation for an evaluation nonetheless. The subjective self-assessment of different skill levels may present a further limitation, though many of these skills prove difficult to assess objectively with simple and fast methods. This was balanced using measurable criteria like years of practice or number of sutures performed in the past 12 months, and performing univariate analysis of all factors due to possible confounding.

The present study provides novel insights into robotic microsurgery, analyzing the largest series of consecutive robotic assisted microsurgical anastomoses performed by participants of different surgical skill levels in a preclinical setting, to our current knowledge. We were able to show a steep learning curve in the whole trial cohort with improved speed and skill at handling the robotic surgical system after only few anastomoses. Surgeons with more surgical and microsurgical practice show higher technical scores and speed in the beginning of the study but the influence of these differences in prior skill levels diminish with escalating case numbers. Though further research is warranted regarding specific areas of application and cost-effectiveness, results of this study should serve as encouraging evidence for the preclinical and clinical implementation of robotic surgical systems like the Symani, as it highlights the rapid improvement of a robotic assisted microsurgical skill set in both novice and advanced surgeons.

## Supplementary Information

Below is the link to the electronic supplementary material.

Supplementary file1 (DOCX 16 KB)

## Data Availability

No datasets were generated or analysed during the current study.
