# Supplementary material for: Learning curve of robotic assisted microsurgery in surgeons with different skill levels: a prospective preclinical study
Source: J Robot Surg. 2024 Sep 28;18(1):353. doi: 10.1007/s11701-024-02114-2 (PMC11438828; doi:10.1007/s11701-024-02114-2)
Supplement: Supplementary file 1 — Supplementary file1 (DOCX 16 KB) [file 11701_2024_2114_MOESM1_ESM.docx]

Journal of Robotic Surgery

**Learning Curve of Robotic-Assisted Microsurgery in Surgeons with Different Skill Levels - A Prospective Pre-Clinical Study**

Donata von Reibnitz^1^, Andrea Weinzierl^1^, Lisanne Grünherz^1^, Pietro Giovanoli^1,2^, *Nicole Lindenblatt^1,2^

*^1^ Department of Plastic and Hand Surgery, University Hospital Zurich (USZ), Zurich, Switzerland*

*^2^ University of Zurich (UZH), Zurich, Switzerland*

*Corresponding Author: nicole.lindenblatt@usz.ch

**Supplementary Material 1** Modified Structured Assessment of Microsurgical Skills (SARMS)

| Category | Skills | 1 | 2 | 3 | 4 | 5 |
| --- | --- | --- | --- | --- | --- | --- |
| Dexterity | *Bimanual dexterity* | Lack of use of non-dominant hand |  | Occasionally awkward use of non-dominant hand |  | Fluid movements with both hands working together |
|  | *Tissue handling* | Frequently unnecessary force with tissue damage |  | Careful but occasional inadvertent tissue damage |  | Consistently appropriate with minimal tissue damage |
| Visuo-spatial ability | *Microsuture placement* | Frequently lost suture and uneven placement |  | Occasionally uneven suture placement |  | Consistently, delicately and evenly spaced sutures |
|  | *Knot technique* | Unsecure knots |  | Occasional awkward knot tying and improper tension |  | Consistently, delicately and evenly placed sutures |
| Operative flow | *Motion* | Many unnecessary or repetitive moves |  | Efficient but some unnecessary moves |  | Economy of movement and maximum efficiency |
|  | *Speed* | Excessive time at each step due to poor dexterity |  | Efficient time but some unnecessary or repetitive moves |  | Excellent speed and superior dexterity without awkward moves |
| Robotic skills | *Depth perception* | Frequent inability to judge object distance |  | Occasional empty grasp |  | Consistently able to judge spatial relations |
|  | *Wrist articulation* | Little or awkward wrist movement |  | Occasionally inappropriate wrist movement or angles |  | Continually using full range of endowrist motion |
|  | *Atraumatic needle and suture handling* | Consistent bending/breakage of needle/suture |  | Occasional bending/breakage of needle/suture |  | Consistently undamaged needle/suture |
|  | *Atraumatic tissue handling* | Consistent inappropriate grasping/crushing or over spreading of tissue |  | Occasional inappropriate grasping/crushing or over spreading of tissue |  | Consistently gentle handling of tissue |
